# Multiple Intestinal Erosions as a Result of Hemorrhage due to Parasites: Case Reports and Review of the Literature

**DOI:** 10.1155/2011/340869

**Published:** 2011-06-01

**Authors:** Hannah Pitanga Lukashok, Carlos Robles-Jara, Carlos Robles-Medranda

**Affiliations:** Gastroenterologia y Endoscopia Division, Instituto Ecuatoriano de Enfermedades Digestivas (IECED), OMNI Hospital, Universidad de Especialidades Espiritu Santo (UEES), Guayaquil, Ecuador

## Abstract

Obscure gastrointestinal bleeding appears to be uncommon in patients with parasites. In spite of that some reports had described this relationship in patients evaluated during capsule endoscopy procedures; the characteristic of the bleeding lesions remains unclear. This paper describes two patients with a massive obscure gastrointestinal bleeding due to ascariasis, using the new capsule endoscopy technology “MiroCam”, describing the characteristic of the lesions found in our patients (observed in a better image quality), and reviewing the literature.

## 1. Case 1

A 54-year-old woman was admitted to our center because of a massive gastrointestinal bleeding (melena). On physical examination the patient was pale, with a pulse rate of 110 bpm and a blood pressure of 90/60 mm Hg. Laboratory results showed a hemoglobin (Hb) of 9 g/dL (*N* = 12–16 g/dL). There was no comorbidities, history of nonsteroidal antiinflammatory drugs (NSAIDS) use, allergies, or clinical-laboratorial parameters of inflammatory bowel disease. 

**Figure 1:**
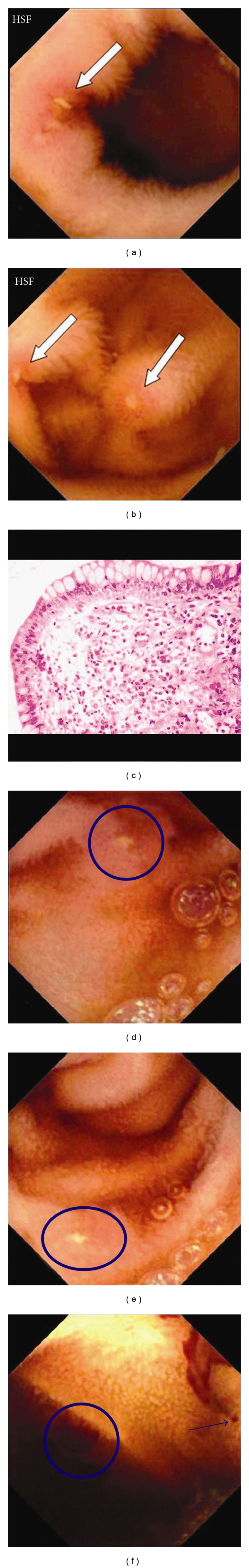
Intestinal erosions with erythema ring and fibrin center point detected by CE: (a) Case 1 at 2 h 50 min; (b) Case 1 at 2 h 54 min; (c) histology of an ileal erosion in Case 1 showing an eosinophil infiltrate with some neutrophils and increase of the mononuclear cells. (d) Case 2 at 9 h 55 min; (e) Case 2 at 9 h 60 min; (f) an intestinal bleeding point at 10 h 02 min in Case 2.

The upper endoscopy was normal. Ileocolonoscopy only showed an erythema ring with a central fibrin point in the terminal ileum that was biopsied. The histology described a moderate eosinophilic infiltrate with some neutrophils and increase of the mononuclear cells (Figure 1(c)). The capsule endoscopy (Figure 1) showed multiple erosions in a total number of 20 with an erythema ring and a central fibrin point from the jejunum to the terminal ileum. The stool test found the presence of ascaris eggs. Treatment consisted of nitazoxanide 500 mg PO bid for 3 days. There was no rebleeding in a followup of 6 months.

## 2. Case 2

A 36-year-old man was hospitalized because of hematochezia, abdominal discomfort, and signs of an important blood loss on the physical exam (pallor, diaphoresis, pulse rate >100 bpm, and blood pressure 100/60 mm Hg). The Hb was 8 g/dL (*N* = 12–16 g/dL). The other laboratory tests were normal. There was no history of diarrhea, abdominal pain, allergies, NSAIDS use, or comorbidities. The gastroscopy and colonoscopy were normal. Capsule endoscopy showed four erosions (Figure 1) in the jejunum (with an erythema ring and fibrin center point) with a bleeding red point in one of them (Figure 1(f)). The stool test found the presence of ascaris eggs. Treatment consisted of blood transfusion and albendazole 400 mg PO with good response.

## 3. Discussion

Capsule endoscopy (CE) is a relatively new tool for evaluating the small bowel [1], being most commonly used to evaluate obscure gastrointestinal (GI) bleeding [2]. The “MiroCam” capsule (Intromedic, 1104 E&C Venture Dream Tower 6-Cha, 197-28 Guro-Dong, Guro-Gu, Seoul 152-719, Republic of Korea) that was used in our patients is a new endoscopy capsule (recently introduced in Ecuador) that uses a novel transmission technology (electric-field propagation). It permits the body to be a conductive medium for data transmission, permitting a better image resolution (320 × 320) and longer battery duration (more than 11 hours) [3]. 

*Ascaris lumbricoides* has been estimated to affect up to 1.5 billion people, largely in tropical and subtropical countries, such as Ecuador. The majority of patients with ascariasis are asymptomatic. However, a minority have pulmonary symptoms during the lung phase of larval migration or gastrointestinal symptoms. 

Gastrointestinal symptoms usually occur in the presence of large worm burdens and include intestinal obstruction, volvulus, obstructive jaundice, cholangitis, cholecystitis, pancreatitis, and bleeding, whether it is occult, obscure, or massive, from the small bowel, the colon, and even the esophagus [4]. Gastrointestinal bleeding appears to be uncommon with ascariasis but might develop as a result of chemical irritation by secretions or by mechanical trauma [5].

However, after the introduction of pushing enteroscopy and capsule endoscopy, some reports have described the intestinal lesions caused by worms, as well as worms themselves. 

There have been four reports in which the presence of *Ascaris lumbricoides* has been described during capsule endoscopy exploration [4, 6–8]. Two cases were reported because of gastrointestinal hemorrhage [4, 6], and only one of them describes an intestinal erythematous point “suggestive” of an angiodysplasia as a cause of the bleeding [6]. 

The diagnosis of ascariasis is usually made by the identification of eggs on stool microscopy. 

On the other hand, the first description of massive jejunal bleeding by ascariasis was performed during enteroscopy investigation [9]. The authors reported multiple rounded or oval erosions with 2–4 mm in size, with presence of fresh blood, oozing from erosions [9]. Lesions were also observed in our cases.

In one of our patients, ileal exploration was possible during colonoscopy. One erythema ring with a central fibrin point was found. Biopsy of this lesion reveals a moderate eosinophilic infiltrate with some neutrophils. 

The intestine affected by ascariasis toxins has been found through histology to produce large numbers of plasma cells and eosinophils [5]. A similar histology was found in one of our patients. 

Treatment should be conservative, with antiparasitic agents and in necessary cases with blood transfusion. 

In our cases, as in cases of massive bleeding by ascariasis, probably the worm presence produced toxins that lead to multiple intestinal erosions as the cause of the bleeding. 

In the near future, the introduction of capsule endoscopy technology in developing countries will detect more lesions of this type that in the presence of a stool test positive for worms as ascariasis, antiparasitic treatment should be started avoiding other unnecessary invasive tests.
